# Extraordinary evanescent field confinement waveguide sensor for mid-infrared trace gas spectroscopy

**DOI:** 10.1038/s41377-021-00470-4

**Published:** 2021-01-29

**Authors:** Marek Vlk, Anurup Datta, Sebastián Alberti, Henock Demessie Yallew, Vinita Mittal, Ganapathy Senthil Murugan, Jana Jágerská

**Affiliations:** 1 Department of Physics and Technology, UiT The Arctic University of Norway, NO-9037 Tromsø, Norway; 2 Optoelectronics Research Centre, University of Southampton, Southampton, SO17 1BJ UK

**Keywords:** Mid-infrared photonics, Optical sensors, Infrared spectroscopy, Imaging and sensing, Integrated optics

## Abstract

Nanophotonic waveguides are at the core of a great variety of optical sensors. These structures confine light along defined paths on photonic chips and provide light–matter interaction via an evanescent field. However, waveguides still lag behind free-space optics for sensitivity-critical applications such as trace gas detection. Short optical pathlengths, low interaction strengths, and spurious etalon fringes in spectral transmission are among the main reasons why on-chip gas sensing is still in its infancy. In this work, we report on a mid-infrared integrated waveguide sensor that successfully addresses these drawbacks. This sensor operates with a 107% evanescent field confinement factor in air, which not only matches but also outperforms free-space beams in terms of the per-length optical interaction. Furthermore, negligible facet reflections result in a flat spectral background and record-low absorbance noise that can finally compete with free-space spectroscopy. The sensor performance was validated at 2.566 μm, which showed a 7 ppm detection limit for acetylene with only a 2 cm long waveguide.

Optical sensors based on infrared tuneable diode laser absorption spectroscopy (TDLAS)^1–4^ are traditionally used for the most demanding applications in trace gas detection, from atmospheric monitoring of climate gases to detecting traces of methane on Mars by NASA’s Curiosity Mars Rover^5^. These sensors rely on strong light absorption at the rotational–vibrational resonance frequencies of gas molecules and long optical interaction pathlengths, leading to detection limits below part-per-trillion (ppt) concentration levels^2^. Although a large variety of system configurations have been demonstrated, the most sensitive systems employ optical multi-pass cells or cavities^2,3^ where the free-space beam is folded into tens of metres to kilometres to increase the probability of absorption by sparse target molecules. However, the large dimensions and sample volumes of multi-pass cells and their incompatibility with large-scale and cost-effective sensor deployment motivate efforts to seek alternatives using integrated photonics^6–11^. Such TDLAS sensors will allow the implementation of long optical pathlengths on chips, thus radically decreasing the instrument size and price, minimizing the sample volume, and relaxing gas flow and temperature control constraints. However, current photonic waveguides still suffer from propagation losses that limit the pathlength to several tens of centimetres. The losses mainly originate from roughness-induced scattering in the near-infrared (NIR) region and from material absorption due to residual impurities, water, OH, or NH^12,13^ in the mid-infrared (MIR) region. Furthermore, reflections at waveguide facets and defects result in distinct etalon patterns in the transmission spectra. This spectral noise interferes with the signal and drastically reduces the gas detection capability and sensor stability^3,8,14^. Finally, yet importantly, the evanescent field in conventional air-cladded waveguides limits the light–matter interaction to a fraction of that for free space. Integrated waveguides must therefore be proportionally longer than the distance travelled by a free-space beam to achieve the same sensitivity.

Light absorption in waveguides exposed to an absorbing environment can be expressed by a generalized Lambert–Beer law $I = I_0\exp [ - \alpha {\Gamma}L]$, where *Γ* represents the external evanescent field confinement factor, *α* is the absorption coefficient of the surrounding environment, and *L* is the physical waveguide length. The confinement factor *Γ* gives a measure of light–matter interaction via the evanescent field^11,15–18^, and it can be expressed as^18^

1 $$\begin{array}{*{20}{c}} {{\varGamma}} = \frac{{n_{\mathrm{g}}}}{{{\mathrm{Re}}\left\{ {n_{{\mathrm{cl}}}} \right\}}}\frac{{\int \int _{{\mathrm{cl}}}\varepsilon \left| {\mathbf{E}} \right|^2dxdy}}{{\int \int _{ - \infty }^\infty \varepsilon \left| {\mathbf{E}} \right|^2dxdy}} \end{array}$$

where *n*_g_ is the group index, *n*_cl_ is the cladding refractive index, *ε*(*x*, *y*) is the permittivity, and **E**(*x*, *y*) is the electric field (Supplementary Information S1). Importantly, the absorption in integrated waveguides is not simply scaled by the modal power fraction residing in the cladding but depends on both the electric field distribution and the waveguide dispersion captured through *n*_g_. The combined effect of field delocalization and dispersion can cause *Γ* to exceed unity^16,17^, facilitating stronger absorption than with a free-space beam.

To date, arguably the most successful realization of an on-chip TDLAS gas sensor has been based on a 10 cm long silicon strip waveguide for methane detection, operating at 1651 nm with *Γ* = 25.5%^8,14^. In this work, a sub-100 ppm limit of detection (LOD) was achieved, mainly limited by etalon fringes and the weak absorption of methane in the NIR. In the MIR, three different waveguide sensors were reported to measure CO_2_ concentrations down to 500 ppm, namely, silicon-on-insulator waveguides with *Γ* = 14%^9^, silicon strip waveguides on Si_3_N_4_ membranes with *Γ* = 19.5%^10^, and free-standing silicon strip waveguides supported by isolated pillars with *Γ* = 44%^11^. Even higher confinement factors have been claimed using slow light photonic-crystal waveguides^6,7,19^; however, these came at the expense of high propagation loss limiting the device length to at best 1.5 mm^7^.

In this work, we propose a waveguide sensor that pushes the confinement factor above 100% by making use of strong guided mode delocalization rather than waveguide dispersion. Our design is based on a free-standing high-aspect-ratio tantalum pentoxide (Ta_2_O_5_) membrane, where lateral confinement is achieved by a shallow rib, as illustrated in Fig. 1a. In the direction perpendicular to the membrane surface, the field distribution is mainly governed by the membrane thickness. Specifically, for transversally magnetic (TM) polarization, the evanescent field fraction increases rapidly with decreasing membrane thickness, and already at T = 500 nm, the weak waveguide dispersion is sufficient to bring the external confinement factor *Γ* above 100% (Fig. 1b and Supplementary S2.1). For Ta_2_O_5_ with a moderate refractive index, *Γ* reaches a maximum of 108% at approximately T = 400 nm, implying that a strong per-length interaction can be reached with a relatively thick and mechanically stable membrane.

**Fig. 1:**
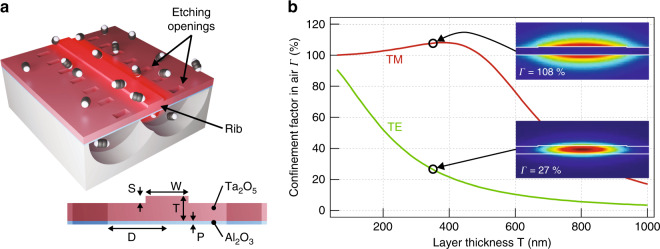
Waveguide design with simulated evanescent field confinement factor in air. **a** Schematics of the free-standing shallow rib waveguide interacting with C_2_H_2_ molecules. The waveguide is patterned in a Ta_2_O_5_ core layer, bottom-passivated by Al_2_O_3_. The membrane is underetched through the etching openings. Dimensions T = 350 nm, S = 30 nm, W = 4.5 µm, D = 25 µm, and P = 30 nm guarantee single-mode propagation at λ = 2.566 µm and negligible mode disturbance by the openings. **b** Simulated confinement factor in air versus Ta_2_O_5_ film thickness T. Insets show |**E** | ^2^ of the fundamental transversally magnetic (TM) and transversally electric (TE) mode field distributions together with air confinement factors for the processed membrane thickness T = 350 nm

To avoid interaction between the evanescent field and substrate in real structures, it is critical to remove the bottom cladding and create a sufficiently large separation between the membrane and the substrate. Oxide films on Si are widely used in microelectromechanical systems (MEMS)^20–23^, and this concept was adopted here, as it allows for deeply underetched, high-aspect-ratio membranes. Ta_2_O_5_ was selected as the core material for its availability in microfabrication process lines, chemical and mechanical stability^24,25^, low thermal expansion^26^, low thermooptic coefficient^27^, and optical transparency spanning from 500 nm to 10 µm^28,29^. A 350 nm Ta_2_O_5_ film was first deposited onto a silicon wafer passivated with a thin layer of aluminium oxide (Al_2_O_3_) to protect Ta_2_O_5_ against undesirable etching. Following a 2-step lithography process to pattern the ribs and etching openings, the membrane was underetched using xenon fluoride (XeF_2_) dry etching. This process resulted in a 130 µm wide membrane with more than 20 μm separation from the Si wafer below, as captured in Fig. 2.

**Fig. 2:**
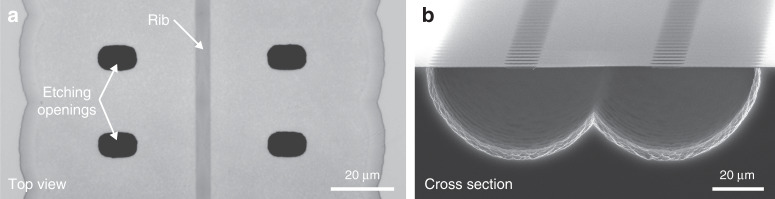
Suspended rib waveguide. **a** Top-view optical microscope image of the fabricated waveguide. **b** SEM image of the waveguide cross section. The fabrication process resulted in a 130 µm wide and 350 nm thick membrane

The waveguides were characterized in an end-fire coupling configuration using the combined imaging and spectroscopy setup depicted in Fig. 3a. A waveguide propagation loss of 6.8 dB cm^–1^ was measured for the TM polarization (Fig. 3b, c), comparable to values reported with other MIR waveguide gas sensors^9,11^. The loss is mainly attributed to light absorption in the Ta_2_O_5_ film due to residual OH and water (Supplementary S5) and is expected to drop significantly with an optimized film deposition process.

**Fig. 3:**
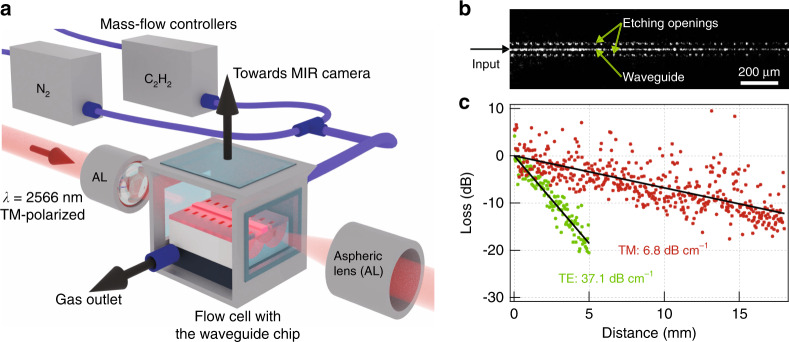
Characterization setup and propagation loss measurement. **a** Outline of the experimental setup. **b** Top-view MIR image of the waveguide at 2.566 µm. The guided mode is visible through out-of-plane scattering at waveguide roughness and imperfections. **c** Propagation loss determined from the decay of out-of-plane scattered light for both TM and TE polarizations. The TE mode that is well confined in the Ta_2_O_5_ membrane experiences higher loss due to material absorption than the strongly delocalized TM mode

For an external confinement factor measurement and a spectroscopy evaluation, acetylene (C_2_H_2_), with its strong Q-band absorption peak near 2.566 µm (3897.1 cm^–1^), was selected as an analyte. Figure 4a compares the experimental transmission spectra measured for a number of C_2_H_2_ concentrations using a 2 cm long waveguide in the TM polarization and a free-space beam of the same pathlength. The experimental spectra were fitted with reference spectra from the Pacific Northwest National Laboratory (PNNL) database^30^ to precisely quantify the amplitude of the absorption peak. As illustrated in Fig. 4b, c, the absorption data acquired over 500 s for different reference gas concentrations between 1% and 10% exhibit good long-term stability and linearity without the need for intermittent recalibration. The fitted concentrations in free space closely match the reference value, but those measured with the waveguide are visibly higher, as they are scaled up by the extraordinary confinement factor. The exact value of *Γ* was retrieved from the ratio of the respective slopes in Fig. 4c, yielding 107 ± 2% in excellent agreement with the simulation.

**Fig. 4:**
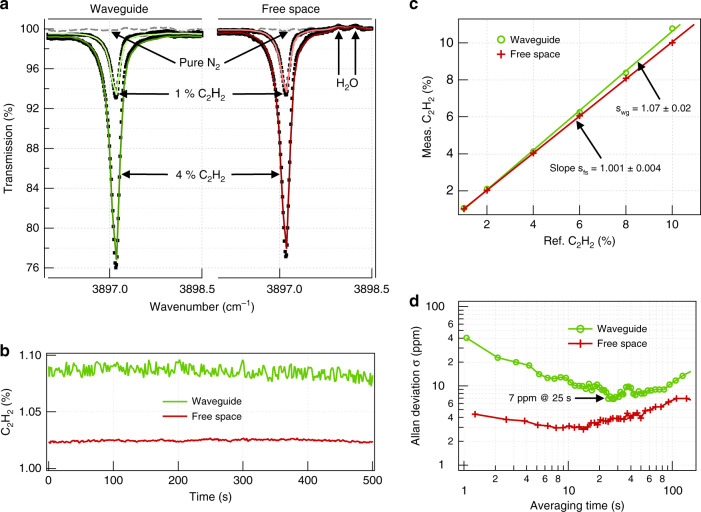
Spectroscopic measurements with acetylene. **a** Transmission spectra (square symbols) and PNNL fits (solid lines) at different concentrations of C_2_H_2_ in N_2_ measured with a 2 cm long waveguide (left) and in free space of equal pathlength (right). The spectra are normalized to the transmittance with dry N_2_ (gray dashed lines). **b** Concentration time series for 1% C_2_H_2_ obtained from real-time spectral fitting with PNNL free-space reference spectra. **c** Measured C_2_H_2_ concentrations versus reference. The ratio s_wg_/s_fs_ yields the evanescent field confinement factor in air as *Γ* = 107 ± 2% free from systematic error that may occur due to uncertainty in the calibration gas concentration or dilution. **d** Allan deviation plot corresponding to the concentration data time series shown in **b**. LOD of 7 ppm after 25 s averaging achieved with the waveguide translates into the absorption noise of 4.5 × 10^−5^

The LOD was evaluated using Allan deviation analysis^4^ with a 1% nominal C_2_H_2_ concentration measured for over 20 min (Fig. 4d). The 1σ-LOD was 7 ppm and 3 ppm using the waveguide and in free space, respectively, with the waveguide data visibly influenced by short-term coupling noise. Hence, despite comparably stronger light–matter interaction in the waveguide, the sensitivity of the free space has not yet been surpassed. However, we stress that this result was achieved with bulk coupling optics, with no thermal stabilization, and without any fringe removal or spectral filtering algorithms, and it is likely to be improved by more elaborate signal processing methods^14^. Nevertheless, the mere 2.5-fold difference in the LOD and comparable system stability almost close the performance gap between the on-chip and free-space-based MIR TDLAS analyzers.

This achievement is largely due to strong field delocalization, which enhances the sensitivity and minimizes the reflections at waveguide facets and defects. Due to a remarkably low effective index of the guided mode (*n*_eff_ ≅ 1.08), reflections at the waveguide facets are ~0.1% (Supplementary S4 and Fig. S6), which is two orders of magnitude less than those for standard SOI waveguides. Similar low reflections are expected at other fabrication imperfections and material defects. As a result, Fabry–Pérot etalons, which substantially limit the performance of other reported waveguide sensors^3,7,14^, are almost entirely suppressed. Strong evanescent field confinement also implies a low interaction of the mode with the waveguide material (Supplementary S5), which relaxes the requirements on material transparency and allows realization of long waveguides in fairly lossy materials. This property is particularly relevant in the MIR, where the optimization of photonic material is still an active research topic^31,32^. Last but not least, strong field delocalization resulting in a fairly large mode area mitigates absorption saturation effects^33^ that can take place under intense irradiation typical for integrated nanophotonic waveguides. As calculated in Supplementary S6, our waveguide can be safely operated with input powers up to 100 mW at 1 bar before the 2.566 μm acetylene absorption line begins to saturate.

In conclusion, we have demonstrated a novel air-suspended Ta_2_O_5_ rib waveguide with a strongly delocalized field, leveraging the unique features of free-space beams for on-chip spectroscopy. Consequently, an absorption noise of 4.5 × 10^–5^ retrieved from the measured LOD was demonstrated^1^. This is, to our knowledge, the lowest value reported for a TDLAS platform based on integrated optical waveguides, either in the NIR or MIR. The current waveguide design curled in a spiral can easily reach 20 cm on a 1 cm^2^ footprint, pushing the LODs for most gases to below part-per-million concentration levels while maintaining a minimal size of the sensor and microlitre sample volumes. Although on-chip sensors may not beat the kilometre pathlengths and ppt detection limits of high-end free-space TDLAS systems, radical sensor miniaturization will facilitate sensor deployment in process control, climate and space research and may open a whole class of new applications that are still out of reach for laser absorption spectroscopy. Examples include sensor organization in networks or in situ monitoring of metabolic processes in microbiology or organoid research. In the latter case, unprecedently small sample volumes combined with high sensitivity and specificity, inherent to TDLAS detection methods, will, for the first time, allow the quantification of metabolic gas release in situ and, so to speak, at a cellular level. Moreover, parallel waveguides patterned on the same chip, each optimized for a different analyte species, can readily realize a multi-gas sensor, with only a negligible increase in its footprint and complexity.

## Methods

### Waveguide simulations

All simulations were performed with the finite differences eigenmode solver Lumerical, MODE. Perfectly matched layers (PMLs) were used on the bottom and both sides of the computational domain to assess the mode leakage into the substrate and the lateral leakage of the TM mode. The effect of the etching openings on the guided mode was studied by varying the width of the computational domain. Single-mode conditions, complex effective indices, mode distributions, and coupling losses were simulated using built-in functions of the software. The confinement factor was calculated using a custom script integrated in the software. More details on the waveguide loss simulation and confinement factor calculation are provided in Supplementary Information S1 and S2.

### Waveguide fabrication

Thin films of SiO_2_ (30 nm), Al_2_O_3_ (30 nm), and Ta_2_O_5_ (350 nm) were deposited in this order onto 4-inch Si wafers by magnetron RF sputtering (Oxford Instruments PlasmaLab 400 +). SiO_2_ promotes the adhesion of Al_2_O_3_ on Si, and Al_2_O_3_ acts as a passivation layer to protect Ta_2_O_5_ during membrane underetching. The deposited films were annealed in a tube furnace at 600 °C in O_2_ for 3 h (1 °C/min ramping) to improve stoichiometry, and reduce the content of residual water and OH in the film.

Waveguides and etching openings were patterned via UV photolithography in two separate steps. For the waveguides, we applied a positive photoresist and exposed the waveguide patterns from a chromium hard mask in a mask aligner (Süss MA-6, λ = 385 nm). Pattern transfer from the resist to the Ta_2_O_5_ layer was performed via Ar ion beam milling (Oxford Instruments Ionfab 300 +). The same procedure was followed for making the etching openings (nominal dimensions 5 × 10 μm^2^, Fig. 2a) with another Cr hard mask aligned to the waveguide pattern. It is critical to expose Si in the etching openings by Ar milling before the next step. Underetching, i.e., the selective removal of Si was performed by dry etching using XeF_2_ (Xactix) in pulsed mode with 270 cycles of alternating XeF_2_/N_2_ etching (3/2 Torr, 5 s) and N_2_ purging (10 s) steps. Ta_2_O_5_ was protected during the release process by photoresist on the top surface and Al_2_O_3_ layer on the bottom surface, which are both substantially more resistant to XeF_2_ than Ta_2_O_5_.

### Waveguide propagation losses

The propagation loss measurement was performed in the combined imaging and spectroscopy setup shown in Fig. 3a. An electrically tuneable DFB diode laser (Nanoplus) emitting a maximum of 15 mW at 2.566 μm was used as the light source. The laser polarization was set to either TE or TM using a half-wave plate and a polarizer. The laser beam was end-fire-coupled into the waveguides using an MIR aspheric lens (Thorlabs, black diamond, NA = 0.56). An MIR camera (Telops) was used to image the sample surface and aid the in-coupling. The propagation loss was measured from the decay of light scattered out of plane from the waveguide and recorded with the MIR camera (Fig. 3a). The raw image data taken along the waveguide length were first corrected for background due to thermal noise and spurious laser light, stitched together, and finally averaged over 10 adjacent pixels to give data presented in Fig. 3a (see also Supplementary S5.1 for further details about the data post-processing). Robustness of the loss value obtained by this method was also cross-checked by the cutback method on three different waveguides from the same wafer that yielded the loss of 6.8 ± 0.7 dB cm^–1^.

### Spectroscopic measurements

The 2 cm long waveguide chip was enclosed in a 2.4 cm long custom gas flow cell with glass windows (Fig. 3a) to provide a controlled environment in terms of the gas concentration and pressure. The latter was kept at 1 bar throughout the experiment. The light transmitted through the waveguide was collected and focused on a single-pixel MCT photodetector (Vigo PVI-3TE-3.4). A custom LabVIEW program was used to electrically tune the laser emission wavelength by 2–3 nm around the target absorption line and to control synchronous data acquisition with a high-speed field-programmable gate array (FPGA) digitizer. Spectral data were acquired at a rate of 1 kHz and averaged in real time to provide 1 s spectra for further data processing. Each spectrum was first divided by a reference measurement with 100% N_2_ to obtain a flat baseline. Water was included in the fit (Fig. 4a) by modelling the spectrum as a sum of H_2_O and C_2_H_2_ spectral templates because weak interference with atmospheric water from outside the gas cell was observed. The normalized spectra were then fitted with calibrated C_2_H_2_ spectra from the PNNL database to obtain the so-called equivalent concentration *C*_eq_, accounting for the total 2.4 cm length of the cell. *C*_eq_ was subsequently corrected for the 0.4 cm free-space contribution between the waveguide facets and the flow cell windows by solving the equation *C*_eq_ × 2.4 = *C* × 2 + *C*_ref_ × 0.4, where *C* and *C*_ref_ are the waveguide-measured and the reference concentrations, respectively. A linear regression fit of *C* versus *C*_ref_, as shown in Fig. 4c, was used to calculate the slope of the line, which represents the average relation between the measured and reference concentration values. For the free-space measurement, the same procedure was repeated with the gas cell without the waveguide chip and collimation optics.

## Supplementary information

Supplementary information
