# Supplementary material for: Extraordinary evanescent field confinement waveguide sensor for mid-infrared trace gas spectroscopy
Source: Light Sci Appl. 2021 Jan 29;10:26. doi: 10.1038/s41377-021-00470-4 (PMC7843987; doi:10.1038/s41377-021-00470-4)
Supplement: Supplementary file 1 — Supplementary information [file 41377_2021_470_MOESM1_ESM.pdf]

Supplementary Information: Extraordinary evanescent field confinement waveguide sensor for mid-infrared trace gas spectroscopy

Marek Vlk[1], Anurup Datta[1], Sebastián Alberti[1], Henock Demessie Yallew[1], Vinita Mittal[2], Ganapathy Senthil Murugan[2]*, Jana Jágerská[1]*

1. Department of Physics and Technology, UiT The Arctic University of Norway, NO-9037 Tromsø, Norway

2. Optoelectronics Research Centre, University of Southampton, Southampton, SO17 1BJ, UK

[1]*jana.jagerska@uit.no, [2]*smg@orc.soton.ac.uk

Contents

S1) Confinement Factor **2**

S2) Design optimization **2**

S2.1) Evanescent field confinement factor in air 3

S2.2) Lateral leakage of TM mode in rib waveguides 4

S2.3) Single-mode condition 5

S3) Spectral response of the waveguide **5**

S4) Etalon effects **6**

S5) Waveguide loss **6**

S5.1) Propagation loss measurement and data post-processing 6

S5.2) Ta_2O_5 bulk loss 7

S6) Saturation of Lambert–Beer absorption **8**

References **9**

S1) Confinement Factor

In optical waveguides, the confinement factor Γ represents a measure of interaction of the guided mode with the waveguide core or cladding, or, more generally, any material constituting the waveguide. Gain or loss α_{m} of a waveguide mode can be obtained from confinement factors Γ_i and bulk gain/loss coefficients α_i of individual materials which constitute the waveguide as

$$\alpha_{\mathrm{m}} = \sum_i \Gamma_i \alpha_i \,. \tag{S1}$$

Light absorption or gain along the waveguide can be then expressed by the generalized Lambert–Beer law

$$I = I_0 \exp\left[-\sum_i \Gamma_i \alpha_i \, L\right] \tag{S2}$$

Particular expressions for the confinement factor have been derived from the Poynting theorem[1] or from the variation theorem for dielectric waveguides[2] and hold for both gain and lossy media such as molecular gases.[3]

Using the Poynting theorem, Visser et al.[1] have found that the confinement factor is given as

$$\Gamma_i = \frac{c\varepsilon_0 \mathrm{Re}\{n_i\} \iint_i \left|\tilde{\mathbf{E}}\right|^2 dxdy}{\mathrm{Re}\{\iint_{-\infty}^{\infty} \left(\tilde{\mathbf{E}} \times \tilde{\mathbf{H}}^*\right) \cdot \mathbf{e}_z dxdy\}}. \tag{S3}$$

with c being the vacuum velocity of light, ε_0 the permittivity of vacuum, n_i the refractive index of ith material, **E** and **H** the electric and magnetic field intensity respectively, and $\mathbf{e}_z$ the normal vector in the direction of propagation, z. Robinson et al.[2] have derived (S3) from the variational theorem for dielectric waveguides.[4] In addition, they have shown that it can also be expressed as

$$\Gamma_i = \frac{n_{\mathrm{g}}}{\mathrm{Re}\{n_i\}} \frac{\iint_i \varepsilon \left|\tilde{\mathbf{E}}\right|^2 dxdy}{\iint_{-\infty}^{\infty} \varepsilon \left|\tilde{\mathbf{E}}\right|^2 dxdy}. \tag{S4}$$

with n_{g} being the group index. The first fraction is linked to the particular material and, more importantly, to the waveguide dispersion. The second fraction represents the normalized electric field energy density in the ith material.

The confinement factor can be readily calculated from (S3) or (S4) for an arbitrary part of the waveguide. In this work, the material of interest was air (particularly a mixture of N_2 and C_2H_2), which constitutes both the top and the bottom cladding of the waveguide (Fig. S1a). In the main text, we therefore reserve the symbol Γ for the evanescent field confinement factor in air.

S2) Design optimization

Fig. S1 schematically portrays the free-standing rib waveguide with its dimensional parameters. When designing the waveguide, we focused on the layer thickness T, the rib width W, and

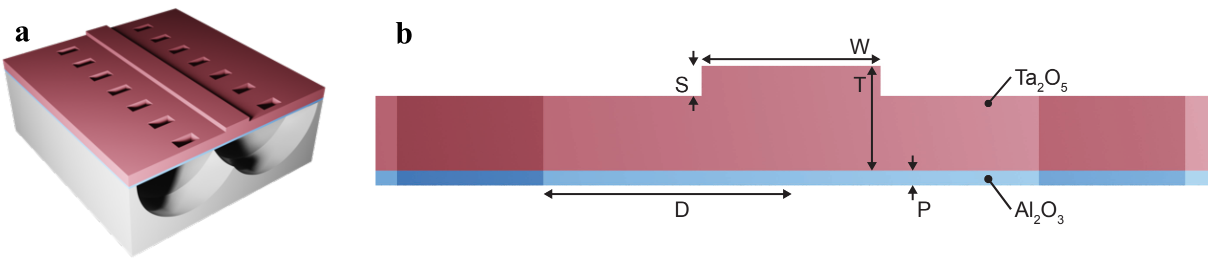

Fig. S1 | Free-standing rib waveguide schematics. a, Illustration of the waveguide which includes a shallow rib, and etching openings. **b**, The waveguide cross section. Our waveguide consists of a core Ta_2O_5 layer and a thin passivating Al_2O_3 layer, which is a necessary feature for the under-etching step in fabrication.

the rib step S for TM polarization at 2.566 μm and refractive indices 2.1 and 1.65 for Ta_2O_5 and Al_2O_3 respectively. All simulations in the design optimization were done with a finite-difference modelling software (MODE, Lumerical) prior to the fabrication. We considered three principal factors that together determined the waveguide dimensions: the evanescent field confinement factor in air Γ, the lateral leakage of the TM mode which occurs in rib waveguides,[5,6] and the single-mode condition.

S2.1) Evanescent field confinement factor in air

As shown in Fig. 1b of the main text, and also reproduced here in Fig. S2a, the evanescent field confinement factor in air depends strongly on the thickness T of the deposited layer but very weakly on the shallow rib dimensions. The change of the rib width W from 3 to 6 μm induced oscillating variations in Γ within only ± 1 % with T = 350 nm. Moreover, the additional layer of Al_2O_3, which is necessary for the fabrication, has only a small effect on the design.

According to Eq. (S4), the value of Γ results from a combination of field distribution and dispersion. Fig. S2b displays separately these two contributions, that is the electric field energy density $\varepsilon|\mathbf{E}|^2$ and the group index n_g. Multiplying these two quantities yields again the Γ plotted in Fig. S2a. In

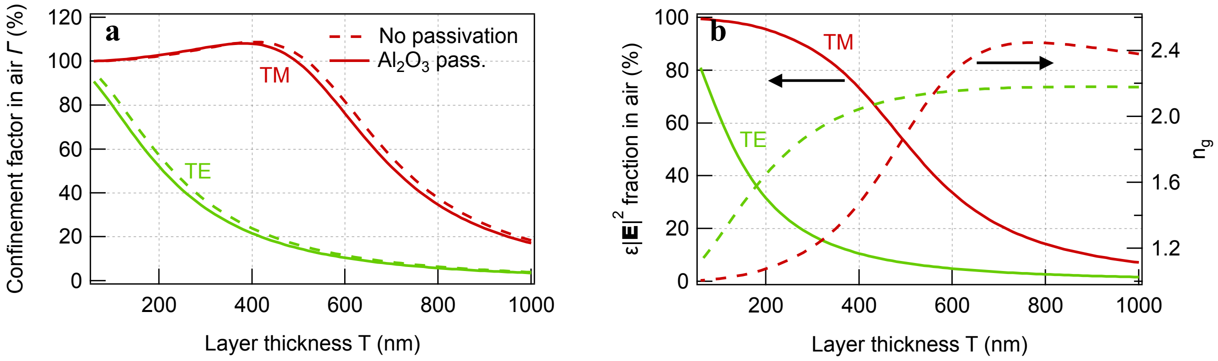

Fig. S2 | Evanescent field air-confinement factor of the free-standing rib waveguide. a, The dependence of the confinement factor on the layer thickness T for both non-passivated Ta_2O_5 membrane and membrane passivated with 30 nm of Al_2O_3. The step S and rib width W were set to 30 nm and 4.5 μm respectively. **b**, Plot of the electric field energy density fraction and the group index. Multiplying these two characteristics yields the confinement factor in air according to eq. (S4).

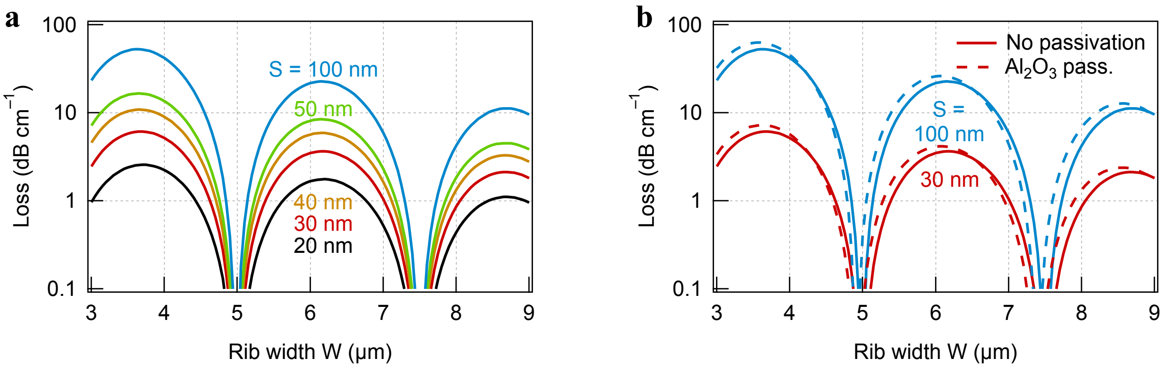

Fig. S3 | Lateral leakage of the TM mode. a, Loss dependence on the rib width for different rib steps from 20 to 100 nm. No Al_2O_3 passivation layer was assumed, and the layer thickness T was fixed to 350 nm. **b**, Deviation in the dependence caused by adding the Al_2O_3 passivation layer.

our waveguide design with T = 350 nm, the major contribution evidently comes from the strongly delocalized field rather than the dispersion, although the combination of the two is required to yield the extraordinary values of Γ. In addition, the extraordinary Γ can be obtained only in TM polarization in the rib waveguide, while for TE polarization, it monotonously increases to unity as the layer is approaching zero thickness.[7]

S2.2) Lateral leakage of TM mode in rib waveguides

The lateral leakage was explained and experimentally demonstrated by Webster et al.[5] It is characteristic only for TM-polarized rib waveguide modes and does not occur for TE-polarized modes. A rib waveguide is naturally formed in a slab which itself is a planar waveguide (see Fig. S1). In essence, the guided TM mode of a rib waveguide couples to a TE planar waveguide mode. This happens for two reasons. First, the guided TM mode has a lower effective index than the TE fundamental planar waveguide mode, which means that there will always be a phase-matched TE mode supported in the slab. Second, like all 2D waveguide modes, the TM rib waveguide mode is hybridized, and so can couple to the TE planar waveguide mode via its minor TE component. As a consequence, the energy of the TM mode is radiated out into the planar waveguide. Nevertheless, this is a resonant effect and the leakage can be avoided if the waveguide dimensions are carefully optimized.

Fig. S3a demonstrates the dependence of the lateral leakage on the rib width W for several step values S, with the layer thickness T fixed to 350 nm. There are pronounced minima in the loss around 5 and 7.5 μm within the studied range of W. We targeted the minimum around 5 μm in our design because such dimension still guarantees single-mode propagation (see the next section S2.3). Although another minimum would occur below W = 3 μm, the corresponding mode would be strongly laterally delocalized, requiring much wider membrane. This would be impractical from the point of membrane fabrication and its resulting mechanical stability.

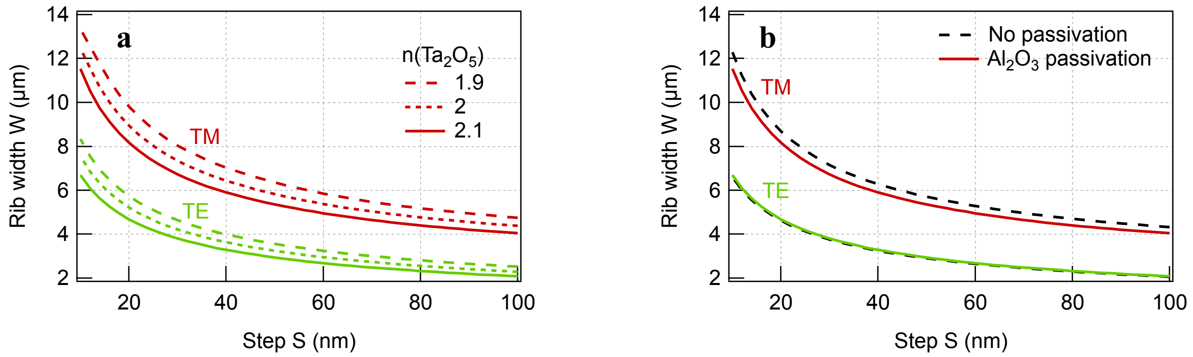

Fig. S4 | Single-mode condition. a, The single-mode condition was studied for several values of the refractive index of Ta_2O_5, which varies depending on the deposition method. The waveguide is single-mode below each curve for the respective polarization and refractive index of Ta_2O_5. Above the curves, the waveguide supports at least one higher order mode. **b**, Influence of the Al_2O_3 passivation layer on the single-mode condition.

Increasing the step S increases the maxima of the loss and narrows the fabrication tolerance for the rib width W. In this work, we selected S = 30 nm as a compromise between the fabrication tolerance and lateral mode confinement which needs to be sufficient in order to avoid scattering on the etching openings. Furthermore, for lithographic tuning, we set the rib width W to 4, 4.5, and 5 µm to follow the lateral leakage loss curve between one maximum and minimum (Fig. S3), and to account for inaccuracies in the fabrication.

Although we present only straight waveguides, the waveguide could be patterned into a spiral to achieve a longer pathlength on a small footprint. E.g. for a limit of a 1 cm^2 chip size, further optimization of parameters S and W would be required after selecting the thickness T.

S2.3) Single-mode condition

We studied the single-mode condition by observing the cut-off for the first-order mode in terms of parameters S and W. Simulations were performed for T = 350 nm and the results are presented in Fig. S4. Any choice of S and W from the parameter space below the plotted cut-off curve will result in single-mode propagation. Hence, step S = 30 nm and rib width W from 4 to 5 µm will yield a single-mode rib waveguide for the TM polarization with a large margin. In the TE polarization, the waveguide already just supports a higher order mode for W = 4 µm.

S3) Spectral response of the waveguide

In Fig. S5, we show how the lateral leakage loss and the confinement factor change in TM polarization with wavelength ranging from 2 to 3 µm. The waveguide maintains leakage loss below 1 dB cm^{-1} from approx. 2300 to 2500 nm, indicating a bandwidth of about 200 nm. It is evident that our current design is not fully optimized for the task of acetylene detection as the theoretical loss is around 1.5 dB cm^{-1} at 2.566 µm. As far as the confinement factor is concerned, the spectral variation within

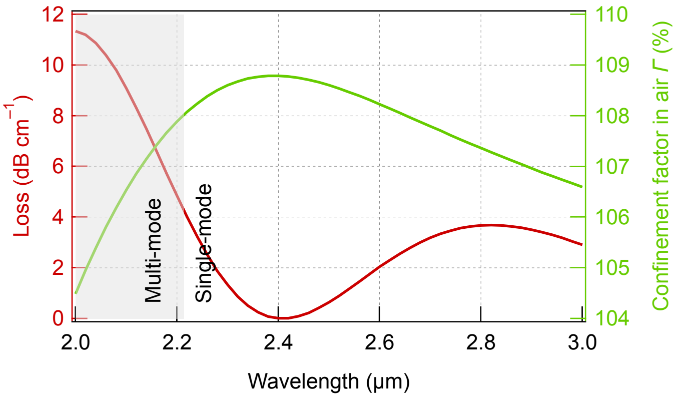

Fig. S5 | Waveguide spectral response. Wavelength dependence of the lateral leakage loss and the confinement factor in air Γ for dimensions T = 350 nm, W = 4.5 μm, S = 30 nm, P = 30 nm, and refractive indices 2.1 and 1.65 for Ta_2O_5 and Al_2O_3 respectively. The shaded area represents multi-mode behaviour.

the 200 nm bandwidth is less than 1 %. The graph also captures the single-mode condition, which occurs at 2.214 μm.

S4) Etalon effects

The TM guided mode exhibits exceptionally low effective index, n_{eff} = 1.08. This translates into low Fresnel reflections between air and the mode at the waveguide facet. In order to quantify the reflection, we used the formula for normal incidence

$$R = \frac{|n_{\mathrm{eff}} - n|^2}{|n_{\mathrm{eff}} + n|^2} \tag{S5}$$

with n = 1 for air, and the n_{eff} data obtained with finite-difference mode solver (MODE, Lumerical). With the dimensions set to S = 30 nm, T = 350 nm, and W = 4.5 μm, we obtained 0.1 % reflection in TM polarization, while TE polarization gave 3.7 % (Fig. S6). Low Fresnel reflection is not only beneficial for the in-/out-coupling, but also helps to minimize etalon effects in the waveguide which otherwise limit the device performance in spectroscopic applications.

S5) Waveguide loss

This section describes the propagation loss analysis. Following the determination of the losses in both TM and TE polarization, and with the knowledge of the core confinement factor we estimated the bulk loss of the tantalum pentoxide.

S5.1) Propagation loss measurement and data post-processing

The loss evaluation was done by analysing MIR camera images of the guided light scattered out of plane. In total, 11 images were acquired for TM polarization and 5 images for TE polarization

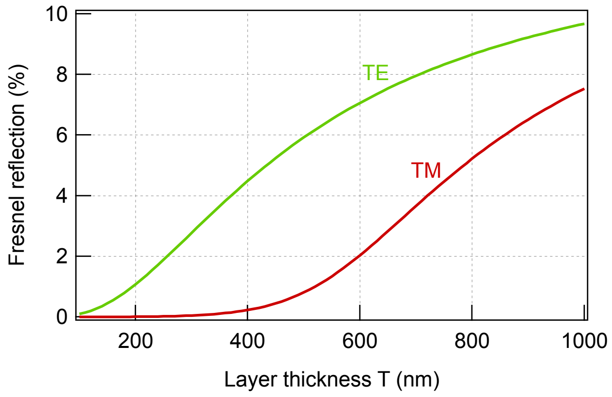

Fig. S6 | Fresnel reflections. Fresnel reflections dependence on a film thickness T for both polarizations. For the calculation, we assumed transmission between the mode of a given effective index and air, and normal incidence.

with a 2 cm long waveguide. The propagation loss in TE polarization is much higher, and there was no appreciable signal after the fifth image corresponding to approx. 1 cm of the waveguide length.

First, these images were corrected for thermal background because the MIR camera captures thermal radiation. This was done by blanking the laser, recording the thermal background, and subtracting it from the signal image. Moreover, the exposure time was individually adjusted for each image as to not saturate the camera.

Subsequently, the images were integrated across the waveguide width, corrected for different exposure times and spurious laser light background, and stitched together to give the complete intensity decay profile as presented in Fig. 3c of the main text (in this Figure, 10-point data averages are shown instead of the full dataset in order to improve the data presentation).

Finally, the intensity profile was converted into decibel (dB) power units as $S_{\mathrm{dB}} = 10\log(S/S_0)$, where S is the intensity value after integrating the camera signal and S_0 the maximum value. The decay profile S_{dB} was fitted with a linear curve, where the slope gave directly the propagation loss.

S5.2) Ta_2O_5 bulk loss

The measured propagation losses in our waveguide are 6.8 dB cm^{-1} in TM and 37.1 dB cm^{-1} in the TE polarization (see Fig. 3c of the main text). These two values together with waveguide loss simulation with complex refractive index allow us to estimate both the bulk absorption loss in the Ta_2O_5 film and the lateral leakage in the TM polarization.

The TE mode loss is assumed to come only from the material absorption. We neglect the scattering loss, which we assume sufficiently low at 2.566 μm as the scattering scales with λ^{-4}. Further, we assume a single-mode TE propagation in this scenario because 1) it is unlikely that the antisymmetric first-order mode will be efficiently excited, 2) due to large lateral extent it will experience strong loss due to the etching openings, and 3) the measured decay is well-described by a single exponential function. By varying the imaginary part of the Ta_2O_5 refractive index,

$n = n' + i\,n''$, we found the best fit with the measured loss value for $n'' = 0.000225$, which corresponds to bulk absorption of 47.9 dB cm^{-1}.

The same n'' yields a loss of 6.1 dB cm^{-1} in the TM polarization, which is in a good agreement with our measurement. The simulation also allows us to discriminate that 4.6 dB cm^{-1} of the total loss comes from the material absorption and 1.5 dB cm^{-1} from the lateral leakage (calculated with $n'' = 0$). The substantially lower absorption loss for the TM mode is a result of weaker confinement in the waveguide core material. Our simulations showed, that the confinement factor in Ta_2O_5 is about 77 % for the TE mode, while it is as low as 9 % for the TM mode.

S6) Saturation of Lambert–Beer absorption

Saturation of linear Lambert–Beer absorption can occur under intense incident irradiation. At sufficiently large intensities, the optical pumping rate on an absorbing transition becomes larger than the relaxation rate, resulting in intensity-dependent decrease of the population in the absorbing level. This manifests as a decrease in absorption coefficient and an additional line broadening.[8] While optical field in free-space beams has a typical mode area of 1–10 mm^2, in photonic waveguides the same optical power is confined to a much smaller area of approx. 0.1–10 μm^2. This results in orders of magnitude higher optical intensities and makes it possible to enter saturation regime at laser powers typical for state-of-the-art laser diodes.

In our experimental case, simulation of the optical field distribution in the waveguide gives a mode area of 3.4 μm^2 with maximum normalized intensity of 1.75×10^{11} m^{-2}. The laser is operated at the power of 15 mW, of which about 8.5 mW is coupled into the waveguide when a measured coupling loss of 2.5 dB is considered. The resulting maximum intensity in the waveguide is 2.5×10^9 W m^{-2}.

If we assume a two-level system dominated by collisional (pressure) broadening, the saturation intensity can be expressed as

$$I_s = \frac{\varepsilon_0 c \hbar^2 \gamma^2}{2\mu^2},$$

Where γ is the pressure-broadened linewidth and μ the dipole moment of the transition.[9,10] μ can be further expressed by the Einstein coefficient A_{ij} and the transition frequency ν_{ij} as[11]

$$\mu^2 = \frac{A_{ij}\, 3\varepsilon_0 h c^3}{16\pi^3 \nu_{ij}^3}.$$

Saturation of Lambert–Beer absorption will occur if the maximum intensity of the guided mode becomes comparable or exceeds I_s. From the HITRAN2016 database,[12] the typical transition dipole moment for lines of the C_2H_2 Q-branch centered at 2.566 μm (3897 cm^{-1}) is $\mu = 4.4 \times 10^{-3}$ D or 1.48×10^{-32} C m and the pressure broadened linewidth at atmospheric pressure 2.7 GHz, giving a saturation intensity $I_S \cong 5 \times 10^{11}$ W m^{-2}.

With maximum intensity in the waveguide of 2.5×10^9 W m^{-2}, still more than 2 orders of magnitude lower than the saturation intensity, saturation of absorption is not occurring at our experimental conditions. This is partly due to the relatively large mode area of our waveguide design resulting from strong optical field delocalization. The absence of saturation was also confirmed experimentally by comparing the lineshapes of the waveguide and the free-space configuration, which appear identical. Nevertheless, saturation may become relevant and the saturation condition must be revisited at lower gas pressures or if detection of gases with distinctly stronger transition dipole moment is targeted.

References

1. Visser, T. D., Blok, H., Demeulenaere, B. & Lenstra, D. Confinement factors and gain in optical amplifiers. *IEEE J. Quantum Electron.* **33**, 1763–1766 (1997).
2. Robinson, J. T., Preston, K., Painter, O. & Lipson, M. First-principle derivation of gain in high-index-contrast waveguides. *Opt. Express* **16**, 16659–16669 (2008).
3. Ottonello-Briano, F. *et al.* Carbon dioxide absorption spectroscopy with a mid-infrared silicon photonic waveguide. *Opt. Lett.* **45**, 109 (2020).
4. Kogelnik, H. Theory of Optical Waveguides. in *Guided-Wave Optoelectronics* (ed. Tamir, T.) vol. 26 7–88 (Springer Berlin Heidelberg, 1988).
5. Webster, M. A., Pafchek, R. M., Mitchell, A. & Koch, T. L. Width Dependence of Inherent TM-Mode Lateral Leakage Loss in Silicon-On-Insulator Ridge Waveguides. *IEEE Photonics Technol. Lett.* **19**, 429–431 (2007).
6. Tsige Dullo, F., Tinguely, J.-C., Andre Solbo, S. & Helleso, O. G. Single-Mode Limit and Bending Losses for Shallow Rib Si_3N_4 Waveguides. *IEEE Photonics J.* **7**, 1–11 (2015).
7. Zhang, X. *et al.* Guiding of visible photons at the ångström thickness limit. *Nat. Nanotechnol.* 1–7 (2019) doi:10.1038/s41565-019-0519-6.
8. Demtröder, W. Widths and Profiles of Spectral Lines. in *Laser Spectroscopy: Basic Concepts and Instrumentation* (ed. Demtröder, W.) 78–114 (Springer, 1981). doi:10.1007/978-3-662-08257-7_3.
9. Castrillo, A., Tommasi, E. D., Gianfrani, L., Sirigu, L. & Faist, J. Doppler-free saturated-absorption spectroscopy of CO_2 at 4.3 μm by means of a distributed feedback quantum cascade laser. *Opt. Lett.* **31**, 3040–3042 (2006).
10. Shimoda, K. Line broadening and narrowing effects. in *High-Resolution Laser Spectroscopy* (ed. Shimoda, K.) 11–49 (Springer, 1976). doi:10.1007/3540077197_18.
11. Absorption and Emission of Light. in *Laser Spectroscopy: Vol. 1 Basic Principles* (ed. Demtröder, W.) 5–60 (Springer, 2008). doi:10.1007/978-3-540-73418-5_2.
12. Gordon, I. E. *et al.* The HITRAN2016 molecular spectroscopic database. *J. Quant. Spectrosc. Radiat. Transf.* **203**, 3–69 (2017).
